# Obituary: in memoriam, Marcel Rudasingwa (18 June 1955 - 17 November 2014)

**DOI:** 10.11604/pamj.2014.19.303.5781

**Published:** 2014-11-20

**Authors:** Robert Davis

**Affiliations:** 1 American Red Cross, International Services, Nairobi, Kenya

**Keywords:** Marcel Rudasingwa, Guinea, UNICEF

## Obituary

**Figure:**
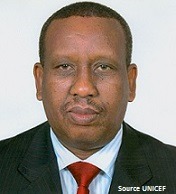


Even by the standards of central Africa, Marcel Rudasingwa lived through turbulent times. During the genocide of 1994, when France looked the other way, Belgium withdrew her forces, and Bill Clinton, keeping his distance from the carnage, inquired about the fate of an attractive Tutsi whom he had met at a reception, Rwanda bled, more than she had ever bled before.

Marcel survived the genocide. So did Monique. Their five children were not so fortunate. With a resilience almost super-human, Marcel and Monique rebuilt their lives, Marcel resuming his work with UNICEF in successive duty postings in Africa and Europe. I remember meeting with him in Bamako a few years back. There was a funding shortfall for the measles campaign. He was all business, pulling out the stops to find the funding needed for a successful campaign. In Nairobi, his last duty posting with UNICEF, he helped mount the successful response against the polio importations of 2013.

On the personal side, he exuded a kind of African warmth mixed with Old World gallantry. He was kind, almost chivalrous, with the opposite sex. Within the UNICEF hierarchy, he managed, as did all of us, within the structures of an organization known for its growing pains over the last few decades. He was no believer in organizational lines. It was by no means surprising that he died while on detachment to the UN, fighting the Ebola epidemic. Here is what I wrote him when I learned of his posting to Guinea.

Dear Marcel, Congratulations or condolences, depending on what you find when you get to Conakry. I can think of no better choice than you. Who else among senior management knows both Guinea and the Supply Division? You are the right man in the right place at the right time. Godspeed, and a safe return!

Cheers, Bob

Read in retrospect, those farewell wishes take on a kind of tragic poignancy. How could such a fine man, a fine rep, a fine husband and father have been cut down in his prime?

Let me give the last word to the book of Ecclesiastes. “I returned, and saw under the sun, that the race is not to the swift, nor the battle to the strong, neither yet bread to the wise, nor yet riches to men of understanding, nor yet favour to men of skill; but time and chance happeneth to them all.”

May he rest in peace.

